# Association of serum intact parathyroid hormone levels with sarcopenia in patients undergoing peritoneal dialysis

**DOI:** 10.3389/fmed.2024.1487449

**Published:** 2024-10-16

**Authors:** Bang-Gee Hsu, Chih-Hsien Wang, Jen-Pi Tsai, Yi-Hsin Chen, Szu-Chun Hung, Yu-Li Lin

**Affiliations:** ^1^Division of Nephrology, Hualien Tzu Chi Hospital, Buddhist Tzu Chi Medical Foundation, Hualien, Taiwan; ^2^School of Medicine, Tzu Chi University, Hualien, Taiwan; ^3^Division of Nephrology, Department of Internal Medicine, Dalin Tzu Chi Hospital, Buddhist Tzu Chi Medical Foundation, Chiayi, Taiwan; ^4^Division of Nephrology, Department of Internal Medicine, Taichung Tzu Chi Hospital, Buddhist Tzu Chi Medical Foundation, Taichung, Taiwan; ^5^Division of Nephrology, Department of Internal Medicine, Taipei Tzu Chi Hospital, Buddhist Tzu Chi Medical Foundation, Taipei, Taiwan

**Keywords:** sarcopenia, parathyroid hormone, atherosclerotic cardiovascular disease, overhydration, peritoneal dialysis

## Abstract

**Objective:**

Sarcopenia is highly prevalent in patients undergoing peritoneal dialysis (PD), contributing to adverse clinical outcomes. Animal models suggest that parathyroid hormone (PTH) induces muscle wasting through adipose tissue browning. However, the relationship between PTH dysregulation and sarcopenia in the PD population remains unclear. Thus, we aimed to explore the association between serum intact PTH levels and sarcopenia in PD patients.

**Methods:**

We conducted a cross-sectional analysis using data from the Tzu-Chi PD cohort, comprising 186 PD patients with a mean age of 57.5 ± 14.1 years. Basic information, comorbidities, serum intact PTH levels, and other biochemical data were retrieved. Atherosclerotic cardiovascular disease (ASCVD) includes any history of coronary artery disease, myocardial infarction, peripheral arterial disease, and stroke. All patients were evaluated for appendicular skeletal muscle mass (ASM) using the Body Composition Monitor (BCM), handgrip strength, and 6-m usual gait speed. Sarcopenia was defined based on the consensus of the Asian Working Group for Sarcopenia 2019. Relative over-hydration (OH) was also assessed using BCM.

**Results:**

The overall prevalence of sarcopenia was 38.2%. Across three groups of intact PTH levels (<150 pg/mL, 150–300 pg/mL, and >300 pg/mL), the prevalence rates of sarcopenia were 29.7, 36.4, and 46.2%, respectively (*p* for trend = 0.044). In the unadjusted model, age, ASCVD, subjective global assessment score, body mass index, relative OH, serum albumin, creatinine, phosphorus, and log-transformed intact PTH levels were significantly associated with sarcopenia. After full adjustment for all above factors, age (odds ratio [OR] = 1.04, 95% confidence interval [CI] = 1.00–1.08), ASCVD (OR = 4.12, 95% CI = 1.34–12.65), BMI (OR = 0.51, 95% CI = 0.41–0.64), relative OH (OR = 1.04, 95% CI = 1.00–1.07), log-transformed intact PTH levels (OR = 3.72, 95% CI = 1.51–9.14) were independently associated sarcopenia among PD patients.

**Conclusion:**

Among PD patients, elevated serum intact PTH levels are independently associated with sarcopenia. Further longitudinal studies are warranted to confirm their causal relationship.

## 1 Introduction

Sarcopenia, characterized by a progressive decline in muscle mass, strength, and physical performance over time (1), significantly increases the risk of falls, fractures, disability, hospitalization, and mortality in patients with end-stage renal disease (ESRD) (2, 3). The prevalence of sarcopenia rises as chronic kidney disease (CKD) advances. Based on a recent meta-analysis from global observational studies, the pooled prevalence of sarcopenia was 16.7% in non-dialysis CKD and further increased up to 35.8% when the disease progressed into ESRD (4). Fully understanding the pathogenesis and mechanisms is crucial to mitigate the progression of sarcopenia in these susceptible patients. Specifically, compared to sarcopenia resulting from aging alone, the mechanisms behind sarcopenia in dialysis patients are far more complex. Apart from well-established factors such as uremic toxins accumulation, protein loss during dialysis, metabolic acidosis, multiple comorbidities, myostatin and angiotensin II overexpression, hyperparathyroidism may also play a pivotal role in the pathogenesis of uremic sarcopenia (5).

Intact parathyroid hormone (PTH) is an 84-amino acid peptide that exerts its biological effects mainly by binding to PTH receptor type 1. The classical effects include enhancing osteoclast activity, inhibiting phosphate reabsorption in the proximal renal tubule, and promoting the conversion of 25-hydroxyvitamin D to 1,25-dihydroxyvitamin D via 1α-hydroxylation in the kidney (6). Elevation of serum PTH universally occurs in advanced CKD and the dysregulation is even exacerbated in ESRD, thus contributing to the central pathogenesis of CKD-mineral and bone disorder (CKD-MBD) (7). Notably, the messenger RNA of the PTH receptor has been identified in skeletal muscle, implicating its potential role in skeletal muscle homeostasis (8). However, the downstream intracellular signaling pathways and their effects on skeletal muscle are still under investigation. Despite this, previous experimental studies have shown that PTH stimulates the release of alanine and glutamine from skeletal muscle and alters muscle metabolism (9), decreases energy production and adenosine triphosphate generation by reducing activities of mitochondrial and myofibrillar creatine phosphokinase (10), enhances resting energy expenditure and induces muscle wasting through adipose tissue browning (11). In ESRD patients who exhibit increased resting energy expenditure (12), elevated serum intact PTH levels have also been linked to greater weight loss (13) and malnutrition (14) among those undergoing hemodialysis (HD). However, no studies have explored the relationship between serum intact PTH levels and sarcopenia in peritoneal dialysis (PD), in which the pattern of serum PTH changes differs from that of HD (15, 16).

Therefore, this study aimed to investigate the association between serum intact PTH levels and sarcopenia among prevalent PD patients.

## 2 Materials and methods

### 2.1 Study design and participants

This cross-sectional analysis was conducted using data from the Tzu-Chi PD cohort, collected between February 2020 and May 2021, across four Tzu-Chi Hospitals in Taiwan, located in Hualien, Chiayi, Taichung, and Taipei. Patients aged >20 years who had undergone PD for more than 3 months were invited to participate, with exclusion criteria including acute infection, active malignancy, the presence of a pacemaker or defibrillator, limb amputation, bedridden status, and refusal to participate. Electronic medical records were utilized to collect basic demographic data, PD duration, and modality, drugs used, as well as medical histories, including diabetes mellitus (DM), hypertension, hyperlipidemia, and atherosclerotic cardiovascular disease (ASCVD), which encompasses any history of coronary artery disease, myocardial infarction, peripheral arterial disease, and stroke. Approval for the study was obtained from the Institutional Review Board of Tzu Chi Hospital (IRB 108-219-A), and all participants provided written informed consent following the principles of the Declaration of Helsinki.

### 2.2 Assessment of appendicular skeletal muscle mass, handgrip strength, and physical performance

In a supine position, a portal whole body multifrequency bioimpedance spectroscopy device (BCM, Fresenius Medical Care, Bad Homburg, Germany) was utilized to assess skeletal muscle and fat tissue mass. This device measures impedance spectroscopy at 50 frequencies and has been extensively employed in evaluating body composition in CKD and dialysis patients, where measurements are less influenced by hydration status (17). Appendicular skeletal muscle mass (ASM) was calculated using the formula: ASM (kg) = −1.838 + 0.395 × total body water (L) + 0.105 × body weight (kg) + 1.231 × male sex −0.026 × age (years). This ASM equation demonstrated an excellent correlation (*R*^2^ = 0.914) with ASM measured by dual-energy X-ray absorptiometry in a Taiwanese dialysis cohort (18). Appendicular skeletal muscle index (ASMI) and fat tissue index (FTI) were determined by dividing ASM (kg) and fat tissue mass (kg) by height squared (m^2^), respectively.

Handgrip strength (HGS) was assessed utilizing a handheld dynamometer (Jamar Plus Digital Hand Dynamometer, SI Instruments Pty Ltd., Hilton, Australia). Patients were positioned standing and instructed to firmly grip the dynamometer, with their arm at a right angle and the elbow positioned alongside the body. Three measurements were taken for each hand with a 1-min rest interval, and the average value of both hands was used for analysis (19).

The usual gait speed (GS) of patients was measured by walking at their natural pace along a flat and straight 6-m path with a static start, and GS was calculated accordingly. Eighteen patients unable to walk were not measured for GS and were classified as having slow GS (19).

### 2.3 Sarcopenia definition

The diagnosis of sarcopenia in this study was based on the consensus of the Asian Working Group for Sarcopenia 2019, which included low ASMI (<7.0 kg/m^2^ for males, <5.7 kg/m^2^ for females) as an essential criterion, along with either low HGS (<28 kg for males, <18 kg for females) or slow GS (<1.0 m/s in both genders) (20).

### 2.4 Subjective global assessment

The Subjective Global Assessment (SGA) was utilized for overall nutritional evaluation, encompassing seven domains: weight change, dietary intake, gastrointestinal (GI) symptoms, functional capacity, comorbidities, subcutaneous fat, and signs of muscle wasting. Each component received a score ranging from 1 (normal) to 5 (very severe), with the total score ranging from 7 (normal) to 35 (severely malnourished) (21). Among these domains, weight change over the past 6 months was scored from no change or weight gain (score = 1) to weight loss greater than 15% (score = 5). Dietary intake was assessed from no change (score = 1) to starvation (score = 5). GI symptoms were scored as follows: 1 = no symptoms, 2 = nausea, 3 = vomiting/moderate symptoms, 4 = diarrhea, and 5 = severe anorexia. Functional capacity was rated from full independence to being bed or chair-ridden with little or no activity. The comorbidities domain considered both the duration of dialysis and the severity of comorbidities. Decreased subcutaneous fat was evaluated based on visible reductions in cutaneous fat below the eyes, on the triceps, biceps, and chest wall. Muscle wasting was evaluated based on inspection of muscle mass in areas such as the temples, clavicles, scapulae, ribs, and quadriceps. The fat and muscle assessment used a scale of 1 for no change, 3 for moderate reduction, and 5 for severe reduction (Supplementary Table 1).

### 2.5 Measurement of serum intact PTH and other laboratory data

Fasting blood samples (~5 mL) were obtained and promptly centrifuged for biochemical analysis. Intact PTH level was measured using enzyme-linked immunosorbent assays (IBL International GmbH, Hamburg, Germany). Two CKD-MBD biomarkers, fibroblast growth factor 23 (FGF23; C-terminal, Immutopics, Inc., San Clemente, CA), soluble α-klotho (Immuno-Biological Laboratories Co., Ltd., Fujioka-Shi, Gunma, Japan), were also measured. Serum levels of creatinine, glucose, albumin, total calcium, and phosphorus, which were routinely measured (Siemens Advia 1800, Siemens Healthcare GmbH, Henkestr, Germany), were retrieved. Corrected calcium levels, calculated as total calcium (mg/dL) + 0.8 [4 – serum albumin (g/dL)], were adopted for analysis. To calculate the total fractional clearance index for urea (Kt/V), 24-h urine and dialysate samples were collected. Individuals with residual urine output were categorized as having preserved residual renal function.

### 2.6 Statistical analysis

The continuous variables were presented as either mean ± standard deviation or median (interquartile range), depending on the normality of the data, as tested by the Kolmogorov–Smirnov test. Categorical variables were presented as absolute numbers (*n*) and relative frequencies (%). The differences between the sarcopenia and non-sarcopenia groups were assessed for significance using either the independent t-test or the Mann–Whitney U test for continuous variables, and the *χ*^2^ test or Fisher’s exact test for categorical variables. Risk factors associated with sarcopenia were screened using univariate logistic regression, selecting variables with significance between the sarcopenia and non-sarcopenia groups, as well as relevant clinical risk factors. The independence of these risk factors was confirmed using multivariate logistic regression, with adjustments made for all significant factors identified in the univariate model. Furthermore, the association between serum PTH levels and sarcopenia was analyzed by categorizing PTH levels into three groups (<150 pg/mL, 150–300 pg/mL, and >300 pg/mL) (22) and evaluated for non-linear relationships using restricted cubic spline analysis. The associations of PTH with individual components of sarcopenia were also explored. Finally, a sensitivity analysis was conducted to assess the robustness of the results by applying the revised European Working Group on Sarcopenia in Older People (EWGSOP2), which represents another relevant update in sarcopenia diagnosis. In EWGSOP2, sarcopenia is defined as low ASMI (<7.0 kg/m^2^ for males, <6.0 kg/m^2^ for females) combined with either weak HGS (<27 kg for males, <16 kg for females) or slow GS (≤0.8 m/s in both genders) (23).

## 3 Results

A total of 186 PD patients, with a mean age of 57.5 ± 14.1 years and a median PD duration of 45 (19–76) months, were included in this analysis. Clinical characteristics of the 186 PD patients, with or without sarcopenia, are summarized in Table 1. Among them, 46.2% were male, 40.3% had DM, 78.0% had HTN, 40.3% had hyperlipidemia, and 19.4% had ASCVD. Sarcopenia was present in 71 (38.2%) patients. Compared to patients without sarcopenia, those with sarcopenia were older and had a higher prevalence of ASCVD. They also had lower height, weight, BMI, ASMI, FTI, HGS, and GS, but higher relative OH. The SGA scores were higher in those with sarcopenia, primarily due to the domains of dietary intake, functional capacity, decreased subcutaneous fat, and signs of muscle wasting (Supplementary Table 1). Regarding laboratory data, patients with sarcopenia had lower serum albumin, creatinine, and phosphorus levels, but higher levels of intact PTH.

**Table 1:** Clinical characteristics of 186 PD patients, with or without sarcopenia.

Characteristics	All patients (*n* = 186)	Sarcopenia (*n* = 71)	No sarcopenia (*n* = 115)	*p*
**Basic demographics**				
Age (years)	57.5 ± 14.1	61.8 ± 16.2	54.9 ± 12.0	0.002*
Gender (male)	86 (46.2)	31 (43.7)	55 (47.8)	0.580
PD duration (months)	45 (19–76)	42 (20–81)	47 (18–73)	0.532
Modality, *n* (%)				
CAPD	97 (52.2)	38 (53.5)	59 (51.3)	0.180
APD or CCPD	89 (47.8)	33 (46.5)	56 (48.7)	
Residual renal function, *n* (%)	112 (60.2)	45 (63.4)	67 (58.3)	0.488
**Diseases, *n* (%)**				
DM	75 (40.3)	23 (32.4)	52 (45.2)	0.083
Hypertension	145 (78.0)	58 (81.7)	87 (75.7)	0.335
Hyperlipidemia	75 (40.3)	31 (43.7)	44 (38.3)	0.466
ASCVD	36 (19.4)	20 (28.2)	16 (13.9)	0.017*
**Examination**				
Height (cm)	160.4 ± 8.5	158.3 ± 7.7	161.7 ± 8.8	0.009*
Weight (kg)	64.8 ± 13.7	56.1 ± 10.1	70.2 ± 12.8	<0.001*
BMI (kg/m^2^)	25.0 ± 4.1	22.2 ± 2.8	26.8 ± 3.8	<0.001*
ASMI (kg/m^2^)	6.5 ± 1.3	5.5 ± 0.9	7.1 ± 1.1	<0.001*
FTI (kg/m^2^)	11.1 ± 4.6	10.0 ± 3.8	11.8 ± 4.9	0.011*
HGS (kg)	21.4 ± 8.7	17.2 ± 6.2	24.0 ± 9.0	<0.001*
GS (m/s)^a^	0.90 ± 0.26	0.84 ± 0.26	0.94 ± 0.26	0.017*
Relative OH (%)	6.8 (−0.3 to 13.6)	9.3 (3.3–15.2)	5.5 (−1.6 to 13.0)	0.036*
**SGA score**	11 (9–12)	12 (9–13)	10 (9–12)	0.002*
**Laboratory data**				
Total Kt/V	2.0 (1.7–2.2)	1.9 (1.6–2.2)	2.0 (1.7–2.2)	0.296
Albumin (g/dL)	3.6 (3.3–3.8)	3.5 (3.3–3.7)	3.6 (3.3–3.9)	0.012*
Glucose (mg/dL)	101 (91–124)	100 (91–124)	103 (92–124)	0.690
Creatinine (mg/dL)	10.8 ± 3.0	9.9 ± 2.4	11.4 ± 3.2	<0.001*
Total calcium (mg/dL)	9.5 ± 0.7	9.4 ± 0.8	9.6 ± 0.7	0.054
Phosphorus (mg/dL)	5.3 ± 1.3	5.0 ± 1.4	5.4 ± 1.3	0.034*
Intact PTH (pg/mL)	249 (98–490)	312 (127–581)	207 (82–412)	0.019*
FGF-23 (pg/mL)	672 (181–2,562)	521 (139–1,776)	854 (241–2,872)	0.073
α-Klotho (pg/mL)	599 (382–802)	581 (376–873)	604 (383–778)	0.876
**Medications, *n* (%)**				
ACEIs or ARBs	124 (66.7)	44 (62.0)	80 (69.6)	0.286
Statins	56 (30.1)	21 (29.6)	35 (30.4)	0.901
Calcium carbonate	125 (67.2)	45 (63.4)	80 (69.6)	0.383
Non-calcium binders	25 (13.4)	12 (16.9)	13 (11.3)	0.277
Active vitamin D	45 (24.2)	20 (28.2)	25 (21.7)	0.320

PD, peritoneal dialysis; CAPD, continuous ambulatory peritoneal dialysis; APD, automated peritoneal dialysis; CCPD, continuous cycling peritoneal dialysis; DM, diabetes mellitus; ASCVD, atherosclerotic cardiovascular disease; BMI, body mass index; ASMI, appendicular skeletal muscle index; FTI, fat tissue index; HGS, handgrip strength; GS, gait speed; OH, overhydration; SGA, Subjective Global Assessment; Kt/V, fractional clearance index for urea; PTH, parathyroid hormone; FGF-23, fibroblast growth factor-23; ACEIs, angiotensin-converting enzyme inhibitors; ARBs, angiotensin II receptor blockers. ^a^*n* = 168. *p < 0.05 was considered significant.

**Figure 1:**
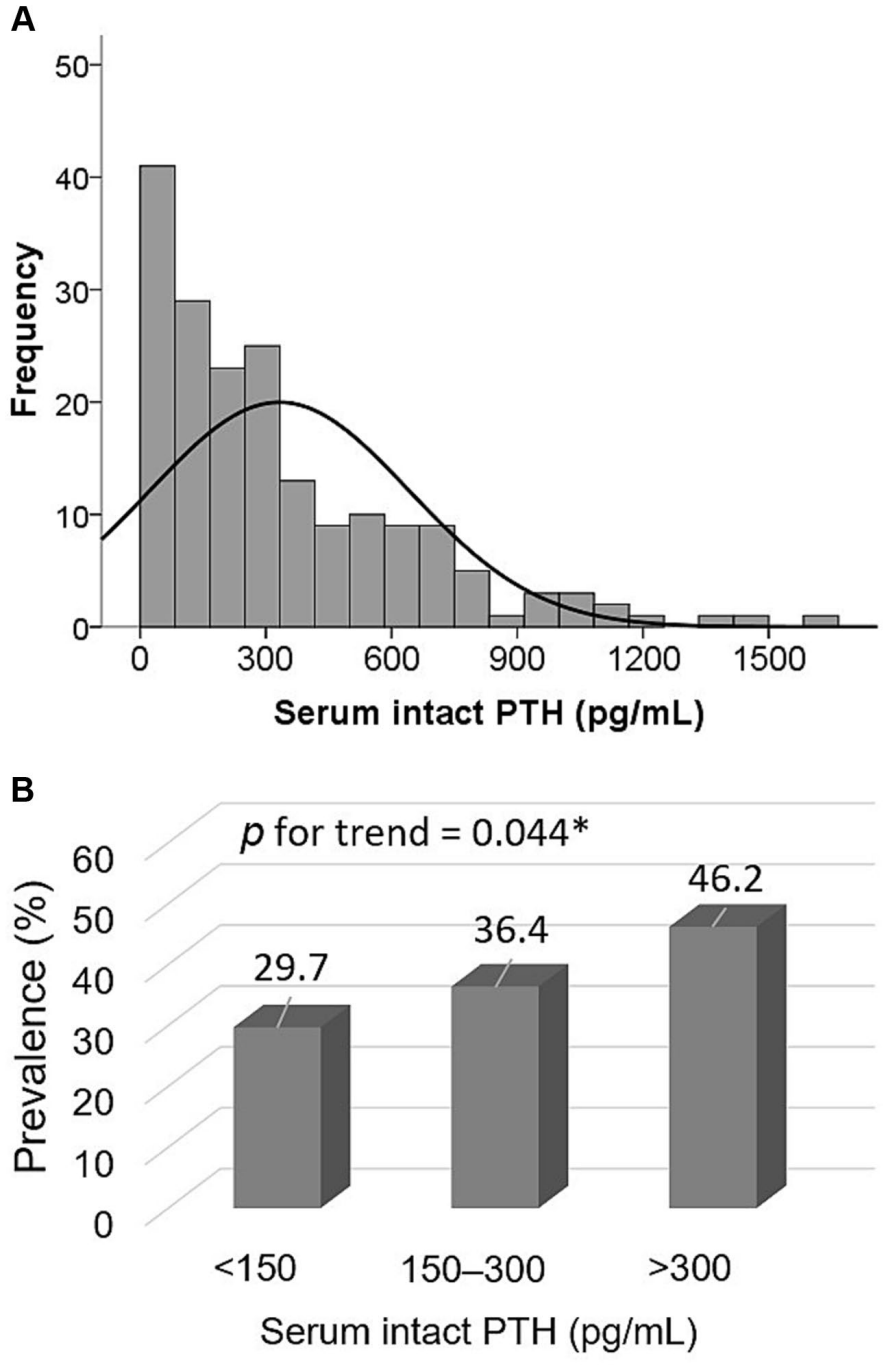
Distribution of serum intact PTH levels (A) and prevalence of sarcopenia among three PTH groups (<150 pg/mL, 150–300 pg/mL, and >300 pg/mL) (B).

The median intact PTH level was 249 (98–490) pg/mL; its distribution is depicted in Figure 1A. Stratified PTH levels into three groups (Figure 1B), those with intact PTH < 150, 150–300, >300 pg/mL had a prevalence of sarcopenia of 29.7, 36.4, and 46.2%, respectively (*p* for trend = 0.044).

Table 2 explores the risk factors associated with sarcopenia among PD patients. In the unadjusted model, age, ASCVD, BMI, relative OH, SGA, serum albumin, creatinine, phosphorus, and log-transformed intact PTH levels were significantly associated with sarcopenia. After full adjustment for all above factors, age (OR = 1.04, 95% CI = 1.00–1.08), ASCVD (OR = 4.12, 95% CI = 1.34–12.65), BMI (OR = 0.51, 95% CI = 0.41–0.64), relative OH (OR = 1.04, 95% CI = 1.00–1.07), log-transformed serum intact PTH levels (OR = 3.72, 95% CI = 1.51–9.14) were independently associated sarcopenia among PD patients. In the sensitivity analysis adopting EWGSOP2 (Supplementary Table 2), the association between log-transformed PTH levels and sarcopenia remained consistent (OR = 3.53, 95% CI = 1.42–8.79).

**Table 2:** Univariate and multivariate factors associated with sarcopenia among PD patients.

Variables	Univariate		Multivariate	
	OR (95% CI)	*p*	OR (95% CI)	*p*
Age (years)	1.04 (1.01–1.06)	0.002*	1.04 (1.00–1.08)	0.035*
Gender (female)	1.18 (0.65–2.14)	0.580	—	—
PD duration (months)	1.00 (0.99–1.01)	0.467	—	—
DM	0.58 (0.31–1.08)	0.085	—	—
ASCVD	2.42 (1.16–5.08)	0.019*	4.12 (1.34–12.65)	0.014*
BMI (kg/m^2^)	0.64 (0.55–0.73)	<0.001*	0.51 (0.41–0.64)	<0.001*
Relative OH (%)	1.03 (1.00–1.05)	0.042*	1.04 (1.00–1.07)	0.029*
SGA score	1.24 (1.09–1.43)	0.002*	1.21 (0.98–1.52)	0.080
Albumin (g/dL)	0.37 (0.16–0.89)	0.027*	1.07 (0.27–4.35)	0.921
Creatinine (mg/dL)	0.84 (0.75–0.93)	0.001*	0.89 (0.73–1.08)	0.236
Phosphorus (mg/dL)	0.78 (0.61–0.98)	0.036*	0.81 (0.53–1.25)	0.340
Intact PTH (pg/mL)^a^	1.85 (1.02–3.35)	0.041*	3.72 (1.51–9.14)	0.004*
FGF-23 (pg/mL)^a^	0.73 (0.48–1.11)	0.139	—	—
α-Klotho (pg/mL)^a^	1.14 (0.30–4.33)	0.850	—	—
Active vitamin D user	1.50 (0.75–3.00)	0.250	—	—

a Log-transformed values.

PD, peritoneal dialysis; DM, diabetes mellitus; ASCVD, atherosclerotic cardiovascular disease; BMI, body mass index; OH, overhydration; SGA, Subjective Global Assessment; PTH, parathyroid hormone; FGF-23, fibroblast growth factor-23. *p < 0.05 was considered significant.

Table 3 analyzes the associations of serum PTH levels and PTH groups with sarcopenia and its individual components. After full adjustment, individuals with intact PTH levels >300 pg/mL exhibited a 4.74-fold increased odds ratio (95% CI = 1.53–14.70) for sarcopenia, compared to the reference group with intact PTH levels <150 pg/mL. Regarding the individual components of sarcopenia, serum PTH levels were significantly associated with low ASMI, but not with low HGS and slow GS. Similar results were observed when applying EWGSOP2 (Supplementary Table 2).

**Table 3:** Association of serum intact PTH levels (continuous and categorical approach) with sarcopenia and its individual components among PD patients.

Intact PTH level	No. of cases	Univariate	Multivariate
		OR (95% CI)	OR (95% CI)
Sarcopenia			
Log-PTH (pg/mL)		1.85 (1.02–3.35)	3.72 (1.51–9.14)
***p***		0.041*	0.004*
<150 pg/mL	64	1 (Reference)	1 (Reference)
150–300 pg/mL	44	1.35 (0.60–3.06)	2.49 (0.71–8.69)
>300 pg/mL	78	2.03 (1.01–4.08)	4.74 (1.53–14.70)
***p* for trend**		0.045*	0.007*
Low ASMI			
Log-PTH (pg/mL)		1.52 (0.88–2.62)	4.52 (1.58–12.96)
***p***		0.136	0.005*
<150 pg/mL	64	1 (Reference)	1 (Reference)
150–300 pg/mL	44	1.11 (0.51–2.42)	1.45 (0.35–5.95)
>300 pg/mL	78	1.54 (0.79–3.00)	3.52 (1.04–11.97)
***p* for trend**		0.200	0.039*
Low HGS			
Log-PTH (pg/mL)		1.18 (0.69–2.02)	1.03 (0.55–1.94)
***p***		0.556	0.925
<150 pg/mL	64	1 (Reference)	1 (Reference)
150–300 pg/mL	44	0.93 (0.42–2.03)	0.82 (0.32–2.08)
>300 pg/mL	78	1.28 (0.64–2.55)	1.22 (0.52–2.84)
***p* for trend**		0.465	0.617
Slow GS			
Log-PTH (pg/mL)		0.79 (0.45–1.38)	0.69 (0.36–1.31)
***p***		0.403	0.251
<150 pg/mL	64	1 (Reference)	1 (Reference)
150–300 pg/mL	44	1.20 (0.53–2.71)	1.36 (0.52–3.52)
>300 pg/mL	78	0.90 (0.45–1.78)	0.77 (0.34–1.78)
***p* for trend**		0.731	0.508

In the multivariate models, age, ASCVD, BMI, relative OH, SGA, creatinine, albumin, and phosphorus were adjusted. *p < 0.05 was considered significant.

**Figure 2:**
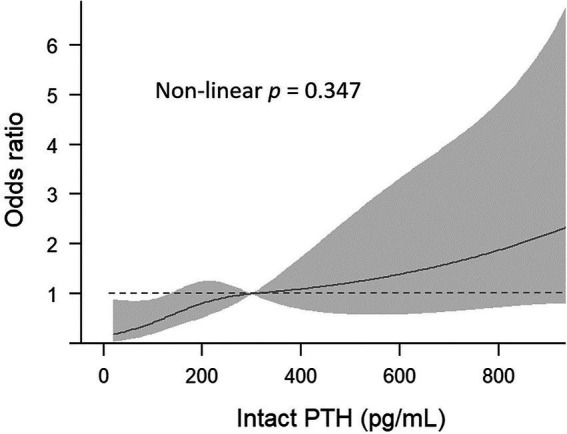
Association of serum intact PTH and sarcopenia, using restricted cubic spline analysis.

Figure 2 illustrates the association between serum PTH groups and sarcopenia through a cubic spline curve. A linear relationship was observed (non-linear *p* = 0.347), indicating that higher intact PTH levels are associated with an elevated risk of sarcopenia.

## 4 Discussion

Our study investigated the serum PTH levels and other relevant risk factors associated with sarcopenia among prevalent PD patients, employing an updated sarcopenia consensus that comprehensively incorporates skeletal muscle mass, strength, and physical performance. The primary finding revealed a linear association between elevated serum PTH levels and increased sarcopenia risk, primarily driven by low ASMI. Furthermore, we identified overhydration and ASCVD as two additional potential modifiable risk factors for sarcopenia among PD patients.

The detrimental effects of PTH on skeletal muscle have been elucidated in animal experimental studies. PTH disrupts the oxidation process of long-chain fatty acids, which serve as a vital energy source for skeletal muscle (24). Specifically, PTH inhibits the activity of carnitine palmitoyl transferase, a key enzyme involved in fatty acid oxidation (24). Another animal study showed that PTH compromises energy production, transfer, and utilization by reducing mitochondrial oxygen consumption, activities of mitochondrial and myofibrillar creatine phosphokinase, and mitochondrial MgATPase (10). More recently, PTH and its associated peptide have been found to induce the browning of white adipose tissue by activating Protein Kinase A signaling and upregulating the expression of uncoupling protein-1, thus increasing basal energy expenditure (11, 25). These effects contribute to skeletal muscle wasting in mouse models of kidney failure and cancer cachexia (11, 25). Similarly, in a mouse model of primary hyperparathyroidism induced by adeno-associated virus injection, adipose tissue browning, increased energy expenditure, and subsequent weight loss have been observed (26). Significantly, both neutralization of PTH-related peptide and knockout of the PTH receptor have shown efficacy in alleviating the loss of muscle mass and strength (25).

The expression of muscle-related mRNAs was altered in patients with primary hyperparathyroidism, including those encoding proteins participating in muscle contracture, regulating myocyte proliferation and differentiation (27). In a previous experimental study, intact bovine PTH and its synthetic 1–34 fragment altered amino acid metabolism of rat skeletal muscle by stimulating the release of alanine and glutamine from muscle in a concentration-dependent manner and may accelerate proteolysis (9). However, whether PTH interplays in the established pathogenesis of sarcopenia and myocyte changes, such as oxidative stress, inflammation, mitochondrial and satellite cell dysfunction, and myostatin overexpression, remains unexplored and should be addressed in further research.

A growing body of clinical evidence indicates the potential adverse effects of elevated PTH levels on skeletal muscle health. In the general population, primary hyperparathyroidism has been associated with increased browning activity, lower body weight, reduced muscle strength, and impaired physical performance (26, 28, 29). Within the ESRD population, individuals with PTH levels ≥1,500 pg/mL showed significantly lower serum albumin and creatinine to body surface area ratio than those with PTH levels between 200–599 and 600–1,499 pg/mL (14). Serum creatinine to body surface area ratio is a simplified muscle index developed in 2014 to replace a reduction of muscle mass in the muscle wasting category of protein-energy wasting initially proposed by the International Society of Renal Nutrition and Metabolism (30). Moreover, individuals in the highest PTH group experienced greater weight loss during longitudinal follow-up (14). A large-scale observational study analyzing data from the Dialysis Outcomes and Practice Patterns Study (DOPPS) showed that HD patients with the lowest PTH levels (e.g., PTH < 50 pg/mL) experienced the least amount of weight loss over 12 months. As PTH levels increased, the amount of weight loss also increased (13). Furthermore, this association between elevated PTH and weight loss partially contributes to diminished patient survival (13). However, direct indicators of muscle health were not evaluated in these studies. Our study, focusing on PD patients, further supported these findings by directly measuring skeletal muscle mass, strength, and physical performance and defining sarcopenia status according to updated consensus guidelines. We found that the association between elevated serum PTH levels and sarcopenia was driven by low skeletal muscle mass.

Although limited by the observational nature of these studies, the detrimental impact of elevated PTH levels on skeletal muscle health in clinical settings is supported by several reports showing improvements in overall nutrition status, skeletal muscle mass, and function following parathyroidectomy in dialysis patients with secondary hyperparathyroidism (31–33). Siqueira et al. reported improved caloric intake, body weight, BMI, phase angle, HGS, SGA score, and reduced weight loss 6 months post-parathyroidectomy (31). Consistently, Jimeno-Fraile et al. observed increased serum albumin, prealbumin, HGS, and improved physical and vitality scores of the 36-items Short Form Health Survey questionnaire (32), and Peters et al. showed increases in phase angle, reactance, and serum albumin levels 6 months post-parathyroidectomy (33).

The optimal range of serum PTH levels in ESRD patients remains uncertain. While the 2003 KDOQI guideline suggests maintaining levels between 150 and 300 pg/mL (22), the 2009 KDIGO guideline proposes a broader range of approximately two to nine times the upper normal limit due to assay variability and limited high-quality evidence linking PTH to CKD-MBD-related outcomes (34). Notably, our study revealed a linear increase in sarcopenia risk with higher serum PTH levels, consistent with findings from the DOPPS analysis where groups with higher PTH levels experienced more significant weight loss over a year (13). In this regard, whether targeting lower PTH levels preserves skeletal muscle mass and function requires further investigation.

In addition to the primary findings, our study yielded some additional insights. We identified ASCVD as a prominent risk factor for sarcopenia among PD patients. Atherosclerosis is widely recognized for its close association with malnutrition in ESRD, primarily mediated by enhanced inflammation (35). Furthermore, atherosclerosis and endothelial dysfunction also contribute to mitochondrial dysfunction in skeletal muscle, impairing vasodilation and subsequently reducing the delivery of amino acids to skeletal muscle (36, 37). Our study unveiled the potential role of ASCVD in sarcopenia, revealing a fourfold increase in the odds of developing sarcopenia among those with ASCVD compared to those without.

Overhydration has been reported to contribute to malnutrition, a decline in muscle mass index, and the development of sarcopenia among PD patients (38, 39), which were in line with our findings. An overhydrated state may adversely impact nutritional status and lead to muscle wasting, driven by chronic inflammation (40), insulin resistance (41), and gut edema (42). However, the causal direction in our cross-sectional study requires further investigation, as patients experiencing nutritional and skeletal muscle loss may struggle to adjust their dry weight promptly, leading to fluid accumulation.

This study is the first to report the association between serum PTH levels and sarcopenia among prevalent PD patients. However, several limitations should be acknowledged. Firstly, despite enrolling PD patients collectively from four hospitals, the sample size was limited. Secondly, given the cross-sectional nature and complexity of CKD-MBD in our study, it is difficult to clarify whether the association between PTH and sarcopenia results from the direct detrimental effect of PTH on sarcopenia or from other CKD-MBD biomarkers influencing serum PTH levels. While we also measured FGF-23 and α-Klotho, other CKD-MBD biomarkers were not fully evaluated. For example, osteocalcin signaling in myofibers has been shown to promote protein synthesis in myotubes without affecting protein breakdown in older mice (43). Thirdly, we utilized the AWGSOP 2019 criteria for identifying PD patients with sarcopenia; unfortunately, there is currently no consensus focusing on the diagnostic criteria for uremic sarcopenia. However, consistent results were achieved when applying EWGSOP2, another updated and relevant sarcopenia consensus. Fourthly, inflammatory markers were not available in this study. Fifthly, the role of overhydration in sarcopenia should be interpreted with caution due to the limitations of the cross-sectional design. Also, ASCVD based on ICD codes could introduce potential non-differential misclassification bias. A more rigorous study design is needed to further explore the interplay between these two factors and sarcopenia. Finally, future research could focus on extending these findings to the broader ESKD population, including patients on HD.

In conclusion, our study highlights elevated serum PTH levels, overhydration, and ASCVD as independent factors contributing to sarcopenia among prevalent PD patients. Beyond well-established strategies for sarcopenia management, including increased daily protein intake and exercise, whether optimizing serum PTH levels, as well as maintaining normal fluid status and addressing ASCVD, prevent the development or mitigate the progression of sarcopenia in PD patients should be investigated in further studies. Moreover, conducting more comprehensive studies is crucial to dissect the role of PTH in sarcopenia. This should involve detailed investigations of myocyte metabolism and clinical muscle indicators, employing a longitudinal design and recruiting a substantial number of participants.

## Data Availability

The raw data supporting the conclusions of this article will be made available by the authors, without undue reservation.
