# Supplementary material for: Association of serum intact parathyroid hormone levels with sarcopenia in patients undergoing peritoneal dialysis
Source: Front Med (Lausanne). 2024 Oct 16;11:1487449. doi: 10.3389/fmed.2024.1487449 (PMC11521897; doi:10.3389/fmed.2024.1487449)
Supplement: Supplementary file 1 [file Data_Sheet_1.docx]

**Supplementary Table 1. The distribution of SGA domains among 186 PD patients, with or without sarcopenia**

| **SGA domains** **score** | **All Patients**  **(n = 186)** | **Sarcopenia**  **(n = 71)** | **No Sarcopenia**  **(n = 115)** | ***p*** |
| --- | --- | --- | --- | --- |
| **Weight change in 6 months, n (%)** |  |  |  |  |
| 1—no weight change or gain | 144 (77.4) | 61 (85.9) | 83 (72.2) | 0.286 |
| 2—weight loss < 5% | 28 (15.1) | 7 (9.9) | 21 (18.3) |  |
| 3—weight loss 5-10% | 9 (4.8) | 2 (2.8) | 7 (6.1) |  |
| 4—weight loss 10-15% | 4 (2.2) | 1 (1.4) | 3 (2.6) |  |
| 5—weight loss > 15% | 1 (0.5) | 0 (0) | 1 (0.9) |  |
| **Dietary intake, n (%)** |  |  |  |  |
| 1—no change | 159 (85.5) | 53 (74.6) | 106 (92.2) | 0.003* |
| 2—sub-optimal solid diet | 25 (13.4) | 16 (22.5) | 9 (7.8) |  |
| 3—full liquid diet | 2 (1.1) | 2 (2.8) | 0 (0) |  |
| 4—hypo caloric liquid | 0 (0) | 0 (0) | 0 (0) |  |
| 5—starvation | 0 (0) | 0 (0) | 0 (0) |  |
| **GI symptoms, n (%)** |  |  |  |  |
| 1—no symptoms | 162 (87.1) | 63 (88.7) | 99 (86.1) | 0.325 |
| 2—nausea | 8 (4.3) | 2 (2.8) | 6 (5.2) |  |
| 3—vomiting or any moderate GI symptoms | 7 (3.8) | 1 (1.4) | 6 (5.2) |  |
| 4—diarrhea | 9 (4.8) | 5 (7.0) | 4 (3.5) |  |
| 5—severe anorexia | 0 (0) | 0 (0) | 0 (0) |  |
| **Functional capacity, n (%)** |  |  |  |  |
| 1—normal | 141 (75.8) | 44 (62.0) | 97 (84.3) | 0.014* |
| 2—difficulty with ambulation | 28 (15.1) | 16 (22.5) | 12 (10.4) |  |
| 3—difficulty with normal activity | 2 (1.1) | 1 (1.4) | 1 (0.9) |  |
| 4—light activity | 3 (1.6) | 2 (2.8) | 1 (0.9) |  |
| 5—bed/chair-ridden with little or no activity | 12 (6.5) | 8 (11.3) | 4 (3.5) |  |
| **Comorbidities, n (%)** |  |  |  |  |
| 1—dialysis <12 months and healthy | 48 (25.8) | 17 (23.9) | 31 (27.0) | 0.852 |
| 2—dialysis 1-2 years or mild comorbidity | 31 (16.7) | 11 (15.5) | 20 (17.4) |  |
| 3—dialysis 2-4 years, age >75 or moderate comorbidity | 46 (24.7) | 17 (23.9) | 29 (25.2) |  |
| 4—dialysis >4 years or severe comorbidity | 61 (32.8) | 26 (36.6) | 35 (30.4) |  |
| 5—very severe multiple comorbidity | 0 (0) | 0 (0) | 0 (0) |  |
| **Decreased subcutaneous fat, n (%)** |  |  |  |  |
| 1—no change | 94 (50.5) | 25 (35.2) | 69 (60.0) | 0.007* |
| 2 | 86 (46.2) | 43 (60.6) | 43 (37.4) |  |
| 3—moderate | 5 (2.7) | 3 (4.2) | 2 (1.7) |  |
| 4 | 1 (0.5) | 0 (0) | 1 (0.9) |  |
| 5—severe | 0 (0) | 0 (0) | 0 (0) |  |
| **Signs of muscle wasting, n (%)** |  |  |  |  |
| 1—no change | 92 (49.5) | 25 (35.2) | 67 (58.3) | 0.006* |
| 2 | 91 (48.9) | 44 (62.0) | 47 (40.9) |  |
| 3—moderate | 2 (1.1) | 2 (2.8) | 0 (0) |  |
| 4 | 1 (0.5) | 0 (0) | 1 (0.9) |  |
| 5—severe | 0 (0) | 0 (0) | 0 (0) |  |

SGA, Subjective Global Assessment; PD, peritoneal dialysis.

**p* < 0.05 was considered significant.

**Supplementary Table 2. Association of serum intact PTH levels (continuous and categorical approach) with sarcopenia and its individual components among PD patients, adopting EWGSOP2**

| **Intact PTH level** | **No. of**  **cases** | **Univariate** |  | **Multivariate** |
| --- | --- | --- | --- | --- |
|  |  | **OR (95% CI)** |  | **OR (95% CI)** |
| **Sarcopenia** | | | | |
| Log-PTH (pg/mL) |  | 2.29 (1.18–4.44) |  | 3.53 (1.42–8.79) |
| ***p*** |  | 0.014* |  | 0.007* |
| < 150 pg/mL | 64 | 1 (Reference) |  | 1 (Reference) |
| 150–300 pg/mL | 44 | 1.34 (0.55–3.26) |  | 2.14 (0.67–6.79) |
| > 300 pg/mL | 78 | 2.48 (1.18–5.23) |  | 4.78 (1.70–13.43) |
| ***p* for trend** |  | 0.014* |  | 0.003* |
| **Low ASMI** | | | | |
| Log-PTH (pg/mL) |  | 1.41 (0.83–2.40) |  | 4.03 (1.36–11.97) |
| ***p*** |  | 0.208 |  | 0.012* |
| < 150 pg/mL | 64 | 1 (Reference) |  | 1 (Reference) |
| 150–300 pg/mL | 44 | 0.91 (0.42–1.97) |  | 0.84 (0.19–3.66) |
| > 300 pg/mL | 78 | 1.23 (0.63–2.38) |  | 1.91 (0.58–6.28) |
| ***p* for trend** |  | 0.525 |  | 0.250 |
| **Low HGS** | | | | |
| Log-PTH (pg/mL) |  | 1.07 (0.63–1.80) |  | 0.93 (0.50–1.72) |
| ***p*** |  | 0.808 |  | 0.818 |
| < 150 pg/mL | 64 | 1 (Reference) |  | 1 (Reference) |
| 150–300 pg/mL | 44 | 1.13 (0.52–2.43) |  | 1.15 (0.47–2.85) |
| > 300 pg/mL | 78 | 1.04 (0.54–2.02) |  | 0.98 (0.44–2.19) |
| ***p* for trend** |  | 0.916 |  | 0.941 |
| **Slow GS** | | | | |
| Log-PTH (pg/mL) |  | 1.32 (0.75–2.33) |  | 1.72 (0.73–4.06) |
| ***p*** |  | 0.330 |  | 0.217 |
| < 150 pg/mL | 64 | 1 (Reference) |  | 1 (Reference) |
| 150–300 pg/mL | 44 | 1.29 (0.58–2.87) |  | 3.24 (0.95–11.02) |
| > 300 pg/mL | 78 | 1.21 (0.61–2.43) |  | 2.01 (0.70–5.71) |
| ***p* for trend** |  | 0.604 |  | 0.280 |

In the multivariate models, age, ASCVD, BMI, relative OH, SGA, creatinine, albumin, and phosphorus were adjusted.

**p* < 0.05 was considered significant.
